# The contribution of family physicians to primary health care: Experiences from southwest Nigeria

**DOI:** 10.4102/phcfm.v13i1.3218

**Published:** 2021-12-17

**Authors:** Temitope Ilori, Kemi T. Awoonidanla, Adedotun A. Adetunji

**Affiliations:** 1 Department of Community Medicine, Faculty of Clinical Sciences, College of Medicine, University of Ibadan, Ibadan, Nigeria; 2 Department of Family Medicine, University College Hospital, Ibadan, Nigeria

**Keywords:** family medicine, primary health care, health equity, comprehensive care, Nigeria, Ibadan

## Abstract

Although an emerging speciality in Africa, family medicine contributes significantly to African health systems. Leadership from family physicians can enable the delivery of high-quality primary health care that is accessible, comprehensive, coordinated, continuous and person-centred. This short report chronicles how family physicians from a university teaching hospital in Ibadan, Nigeria, adopted a health post located in a home for persons with mild physical and mental disabilities and changed it into a hub of comprehensive, holistic and person-centred care for residents and staff of the home, as well as individuals and families in the neighbouring communities and its environs. The Department of Family Medicine of the University College Hospital, Ibadan, with the aid of a benefactor, reorganised a modest health facility to include the services of family medicine senior registrars (undergoing training-related rotations) with supervision by consultant family physicians. Family physicians led the primary health care team that provided both facility-based care and community outreach services. This report demonstrates how family physicians can improve the quality of primary health care and outcomes such as health equity in the community served.

## Introduction

In the African context, the family physician is a clinical leader and consultant in the primary health care team, ensuring primary, longitudinal, comprehensive, holistic and personalised care to individuals, families and communities.^1,2,3^ The speciality of family medicine is relatively new on the African continent. Although there are more family physicians available in practice now than there were some 20 years ago, the speciality is still carving out a niche for itself.

Oluyole Cheshire Home (OCH), Ibadan, established in June 1959 by the Leonard Cheshire disability organisation was the first in-country Cheshire Home to provide shelter and rehabilitation support for disabled persons in Ibadan, Oyo state. In 1980, at the request of the matron in charge, the Oyo State Ministry of Health provided a single-room health post (maintained by two general duty nurses) to cater for the health needs of residents and staff of OCH. This provision was, however, insufficient to meet the basic healthcare needs of residents, and the need for non-governmental support became increasingly obvious as the population of residents (mostly indigent) grew.

Subsequently, a local philanthropist (Mrs Sherifat Kola-Daisi) took the initiative to bridge this gap by donating a purpose-built, on-premise health centre to OCH, and significantly subsidising the cost of healthcare provided at the health centre (so-named Agbeke Mercy Medical Centre [AMMC]). The AMMC debuted in 2008 as a free-of-charge primary care service for residents of OCH with health personnel provided by the Oyo State government. The emergence of affordable healthcare for disabled residents brought on the challenge of accommodating affordable healthcare for their families and needy members of the community surrounding OCH.

It, therefore, became apparent to local administrators over a few years that the aspirations of OCH to support staff, families and community helpers working with disabled residents to raise the standards of care and rehabilitation provided and to adapt interventions to suit local sensibilities would require multidisciplinary collaboration. The need for versatile clinicians with relevant training and contextual competence to manage multiple domains of health for disabled persons and to integrate their care with that of the community in which OCH is located was also established.

In 2010, a stakeholders’ meeting culminated in a multilateral agreement between the Board of Management of OCH, Oyo State Ministry of Health and University College Hospital (UCH), Ibadan, to introduce the services of public health nurses and family physicians who are skilled in the principles and practice of holistic, person-centred/family-centred care at AMMC. The University College Hospital, Ibadan is Nigeria’s premier university teaching hospital with residency training programmes in 32 surgical and medical specialities (including family medicine). Hence, in 2011, the Department of Family Medicine, UCH Ibadan, deployed to AMMC, the first of a long line of family medicine trainees (senior registrar) assigned to complete a supervised 12 week posting in community-based care for vulnerable populations. One of the authors of this report was the pioneer senior registrar at AMMC. This arrangement initiated a sustainable programme to give family medicine resident doctors the opportunity for community-based team leadership and hands-on experience in health administration. At the same time, supporting a local healthcare team to upgrade primary care services available to clients (including preventive care, health screening, youth-friendly sexual and reproductive services, behaviour change communication for health-focused lifestyle modification, point-of-care laboratory services, outpatient medical treatment, care of vulnerable patients including disabled or elderly persons, day case surgery, rehabilitation and social services).

For 10 years (2011 till date), family physicians (supervising consultants and resident doctors) in service at AMMC have continually served advisory roles to the joint board of management of OCH and AMMC. They have led the healthcare team to implement healthcare delivery decisions made by the management board (made up of representatives of stakeholder groups). Residents of OCH have free access to healthcare services (including free medications for treatment), whilst other patients are charged subsidised fees.

## Primary care practice and public health

One of the key interventions involving vulnerable persons attracted to the integrated services provided at the centre is the Older Person Wellness Program (also known as the senior citizens’ club). Older persons living in the neighbourhood of OCH meet once every month at the centre for highly subsidised medical follow-up of patients with non-communicable diseases and peer-group socialising. This intervention was designed to ameliorate this vulnerable group’s lack of access to affordable healthcare by providing cost-effective, person-centred care using a biopsychosocial approach. The senior citizens’ club has motivated regular clinic attendance and good medication adherence amongst elderly patients. Unpublished reports suggest that approximately 80% of people (registered as members of AMMC senior citizens’ club) attend follow-up clinic appointments regularly, with blood pressure (BP) control being optimal in up to 60% of the patients on treatment for hypertension. This compares well with reports of 35% optimal BP control amongst a similar cohort treated for hypertension in a national survey.^4^ Members of the senior citizens’ club also benefit from the affordable point-of-care tests and regular behaviour change communication for maintaining self-care activities. In a society where only a minority of the population has health insurance, prescription medications are provided to patients at highly subsidised cost through a drug revolving fund maintained for this purpose. The provision of family-centred care has fostered the implementation of domiciliary services (such as home visits) and family-level interventions (such as family conferencing for crisis resolution).

The centre has also been a source of screening and diagnosis of hypertension in the community, working actively to combat the lack of awareness amongst hypertensives. Studies have shown that about a third of hypertensives are unaware of their status.^5,6^ Hence, every adult participant at the medical outreach programmes is screened for hypertension and diabetes mellitus. Also, every adult patient has a BP measurement at each clinic visit, irrespective of the clinical presentation. This helps to breach the low rates of hypertension awareness in the Nigerian population.^6,7^ Similarly, patients who require office procedures and minor day-case surgeries are referred to the centre, and the trainee Family Physicians supervised by their trainers carry out these procedures.

Family physicians are trained to provide healthcare to underserved populations, and these skills were brought to fore at the centre.^2,3^ Community entry and engagement were harnessed through community outreach programmes organised by the team led by family medicine residents and consultants. Such outreaches targeted at traders, artisans, older persons and school children have benefitted many people with health promotion information about good health-seeking behaviour, chronic disease prevention and care, human immunodeficiency virus (HIV) infections, education on risky health behaviours and cancer screening. Regular periodic screenings for obesity, breast and cervical cancer are also offered during community outreaches and are usually preceded by patient education and counselling. Point-of-care cervical cancer screening using the visual inspection with acetic acid method is an adopted strategy the centre uses to screen for cervical cancer disease. Although there has been resistance to cancer screening because of fear and inadequate widespread information amongst Nigerian women,^8^ these community outreaches are being used to dispel such misconceptions.

Several generations of students attending nearby government-owned schools have benefited from informal school health services, including care for acute illness and periodic health screening of both the pupils and staff. Youth-friendly reproductive and sexual healthcare is also offered in the centre.

## Lessons learnt

This report demonstrates how family physicians can improve primary health care services and outcomes such as health equity in the community served through community-based team leadership by providing integrated family-oriented and person-centred, affordable comprehensive care.

## Conclusion

This health centre created an opportunity for family physicians and their trainees to demonstrate the capacity to improve the health care system by providing holistic primary health care, improving access to healthcare and eliminating health disparities in an underserved urban poor population.
